# In Silico Design, Synthesis, and Biological Evaluation of Anticancer Arylsulfonamide Endowed with Anti-Telomerase Activity

**DOI:** 10.3390/ph15010082

**Published:** 2022-01-10

**Authors:** Giulia Culletta, Mario Allegra, Anna Maria Almerico, Ignazio Restivo, Marco Tutone

**Affiliations:** 1 Dipartimento di Scienze Chimiche, Biologiche, Farmaceutiche e Ambientali, Università di Messina, 98166 Messina, Italy; giulia.culletta@unime.it; 2 Dipartimento di Scienze e Tecnologie Biologiche Chimiche e Farmaceutiche, Università degli Studi di Palermo, 90123 Palermo, Italy; mario.allegra@unipa.it (M.A.); annamaria.almerico@unipa.it (A.M.A.); ignazio.restivo@unipa.it (I.R.)

**Keywords:** sulfonamides, arylsulfonamide, anticancer compounds, telomerase inhibitors, structure-based drug design, pharmacophore modeling, docking, molecular dynamics

## Abstract

Telomerase, a reverse transcriptase enzyme involved in DNA synthesis, has a tangible role in tumor progression. Several studies have evidenced telomerase as a promising target for developing cancer therapeutics. The main reason is due to the overexpression of telomerase in cancer cells (85–90%) compared with normal cells where it is almost unexpressed. In this paper, we used a structure-based approach to design potential inhibitors of the telomerase active site. The MYSHAPE (Molecular dYnamics SHared PharmacophorE) approach and docking were used to screen an *in-house* library of 126 arylsulfonamide derivatives. Promising compounds were synthesized using classical and green methods. Compound 2C revealed an interesting IC_50_ (33 ± 4 µM) against the K-562 cell line compared with the known telomerase inhibitor BIBR1532 IC_50_ (208 ± 11 µM) with an SI ~10 compared to the BALB/3-T3 cell line. A 100 ns MD simulation of 2C in the telomerase active site evidenced Phe494 as the key residue as well as in BIBR1532. Each moiety of compound 2C was involved in key interactions with some residues of the active site: Arg557, Ile550, and Gly553. Compound 2C, as an arylsulfonamide derivative, is an interesting hit compound that deserves further investigation in terms of optimization of its structure to obtain more active telomerase inhibitors

## 1. Introduction

The nuclear protein complex, Telomere, defends the terminal ends of chromosomes from degradation, end-to-end fusion, and recombination [1,2,3]. The telomere structure is subject to several changes. After each cell division cycle, the telomere gradually shortens until the chromosomal DNA is exposed, inducing a DNA damage response [4,5]. This event helps to maintain the stability of genetic information and protects the genome in a “time-bomb” manner [6]. When the length of telomeres reaches a critical point, cells reach the cycle of termination, aging, and death [5,6]. Normal cells cannot survive this progressive shortening. Sometimes, cells can extend telomeres by reactivating telomerase activity or through a telomere replacement elongation mechanism (ALT) to help cells survive the crisis [7]. The reactivation of telomerase is observed in 85–90% of human tumor cells [8]. Telomerase is a reverse transcriptase that contains an RNA template (TER) with its binding domain (TRBD) and reverse transcriptase unit (TERT). Telomerase is a challenging but appealing target, because the inhibition of its activity can be achieved by acting at different stages and using various mechanisms [9,10,11]. A first but now less popular used approach is based on inhibiting telomerase access to DNA by stabilizing G-quadruplexes formed by a 3’ DNA overhang, which has the task to block telomere elongation [11,12,13,14,15]. Another approach includes the use of inhibitors of the enzymatic active site of telomerase including antisense compounds to TER [16,17]. But the most promising approach is possibly the use of compounds that are able to block the active center of the enzyme in the catalytic subunit, directly. BIBR1532, which selectively inhibits telomerase activity, was first proposed in 2001 and has been patented as a potential anticancer drug [18,19]. As reported in a recent review, structure-based drug design strategies can be used to design potential inhibitors of the telomerase active site [20]. The first structure showing a co-crystallized inhibitor was published in 2015, revealing the *Tribolium castaneum* full-length catalytic subunit of telomerase in complex with the compound BIBR1532 (PDB 5CQG), which showed that the studied ligand interacts outside of the active center [21]. This experimental crystal structure changed the understanding of the expected mechanism of activity of this compound and possibly other active site-directed molecules. BIBR1532 binds to a highly conserved hydrophobic pocket (FVYL) motif on the outer surface of the thumb domain of telomerase. 

Several other compounds designed using in silico approaches have been tested as potential inhibitors of the catalytic subunit of telomerase such as benzylidene–hydrazone analogs [22], dihydropyrazole [23,24,25,26,27,28,29], dibenzopyrroles [30], flavone pyridines [31], 1,3,4-oxadiazole derivatives [32,33,34,35,36], pyrazole-5-carboxamides and pyrazole-pyrimidines [37], spiroketals [37], celastrol derivatives [38], myricetin derivatives [39], indolyl-2’-deoxynucleotide analogs [40], flavonoid derivatives [41], and chrolactomycin derivatives [42]. Other classes of compounds developed as telomerase inhibitors are reported in two recent reviews [43,44]. Thus, considering the direct inhibition of TERT as the most promising approach for blocking the action of telomerase and the lack of an approved drug (Imetelstat is the only one to reach clinical trials [20])makes the search for new and effective inhibitors a hot topic of the pharmaceutical sciences. To the best of our knowledge, there is just one piece of evidence of arylsulfonamide derivatives (SEW05920) as an inhibitor of the TERT [45]. Exploiting our previous experience and outcomes in the use of computational approaches [46,47,48,49,50], we developed a structure-based computational approach to performing a virtual screening of an *in-house* arylsulfonamides library. Some arylsulfonamides have been identified as hits, also synthesized using green chemistry approaches and tested against three cancer cell lines K-562, HCT-116, and MCF-7. The results determined compound 2C as more active than the reference compound BIBR1532. The identification of this novel scaffold, which will undergo optimization, could help to identify new potent telomerase inhibitors.

## 2. Results

### 2.1. In Silico Modeling and Virtual Screening

**Figure 1:**
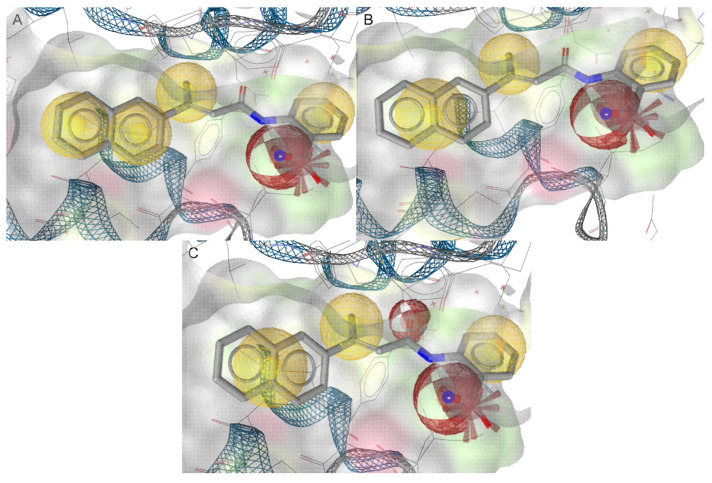
Pharmacophore models derived from the X-ray pose of BIBR1532 (PDB ID: 5CQG): (**A**) static pharmacophore model from the PDB with six features—4 hydrophobic features (yellow spheres), 1 H-bond acceptor (red sphere), and one anionic feature (red star); (**B**) static pharmacophore model after fusing with 5 features—3 hydrophobic features (yellow spheres), 1 H-bond acceptor (red sphere), and 1 anionic feature (red star); (**C**) MYSHAPE pharmacophore model—3 hydrophobic features (yellow spheres), 2 H-bond acceptors (red spheres), and 1 anionic feature (red star).

In this study, we performed a structure-based virtual screening by using MD pharmacophore modeling and docking studies to identify potential telomerase inhibitors in our *in-house* library of arylsulfonamide compounds. Firstly, the *Tribolium castaneum* full-length catalytic subunit of telomerase in complex with the compound BIBR1532 (PDB 5CQG) was selected. The crystal structure was optimized by completing and refining the entire structure and optimizing amide groups of asparagine (Asn) and glutamine (Gln) as well as the imidazole ring in histidine (His); then, we predicted the protonation states of histidine, (His), aspartic acid (Asp), glutamic acid (Glu), and the tautomeric states of histidine. Starting from the PDB coordinates set of BIBR1532, a static pharmacophore model was created by using LigandScout containing six features (Figure 1A):

- Four hydrophobic features;
- One H-bond acceptor;
- One anionic feature.

The pharmacophore features were decreased to five, the two hydrophobic features on the naphthyl ring of BIBR1532 were fused into one, and the tolerance radius for the new pharmacophore feature was increased by 0.15 Å to compensate for small deviations (Figure 1B). To improve the performance of the virtual screening (VS) process, the recent MYSHAPE (Molecular dYnamics SHared PharmacophorE) approach was used [51,52]. According to this approach, the exploration of the protein conformations by molecular dynamics coupled with the pharmacophore modeling improved the result of the VS concerning the corresponding model generated from the PDB coordinates set. To build the MYSHAPE model, 20 ns of molecular dynamics simulation of the BIBR1532–protein complex was run. A new interaction was retrieved such as a hydrogen bond interaction of the carbonyl group of BIBR1532 with the Met482 by a water bridge. The new MYSHAPE pharmacophore feature was added to the original pharmacophore model. In addition, in this case, the tolerance radius for the added pharmacophore feature was increased by 0.15 Å to compensate for small deviations in the 3D coordinates of the different conformations.

The MYSHAPE pharmacophore model had six features (Figure 1C):

- Three hydrophobic features;
- Two H-bond acceptors;
- One anionic feature.

This model was used to screen the *in-house* library of arylsulfonamide derivatives.

**Figure 2:**
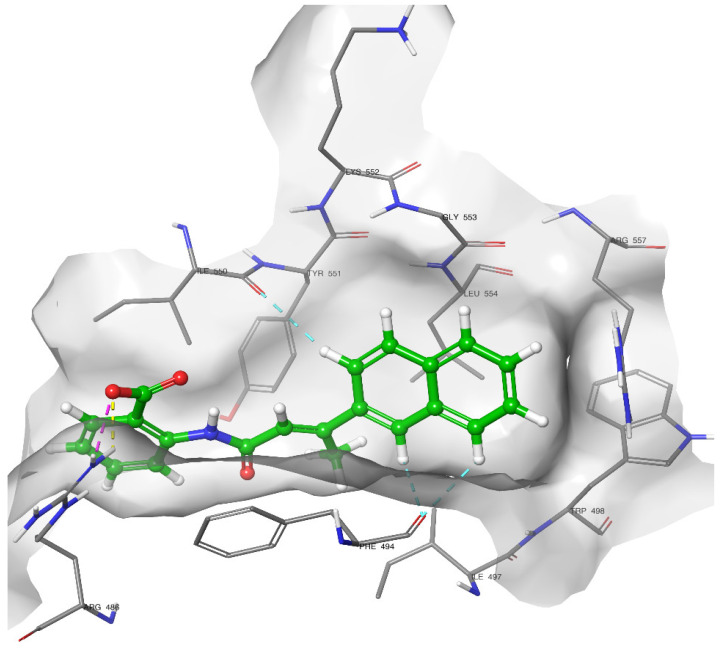
Binding mode after re-docking of BIBR1532: the yellow, dashed lines represent the H-bond interaction and, the purple, dashed lines represent the salt bridge between the O of the carboxyl group and Arg486, and the light blue dashed lines represent the aromatic H-bond interactions between the naphthyl ring and Phe494 and Ile550.

At the same time as the pharmacophore modeling, standard precision (SP) molecular docking studies were performed using Glide [53], considering the conserved hydrophobic pocket (FVYL motif) where BIBR1532 binds. The docking studies were performed centering the docking boxes on the 3D coordinates of BIBR1532. The RMSD of BIBR1532 was calculated showing a value of 0.2 Å. The residues that characterized the binding site were Phe478, Met482, Met483, Arg486, Phe494, Gly495, Ile497, Trp498, Ile550, Tyr551, Gly553, Lys552, Leu554, and Arg557. BIBR1532 established an H-bond interaction and a salt bridge between the oxygen of the amide group and Arg486 and aromatic H-bond interactions between the naphthyl ring and Phe494 and Ile550 (Figure 2).

**Figure 3:**
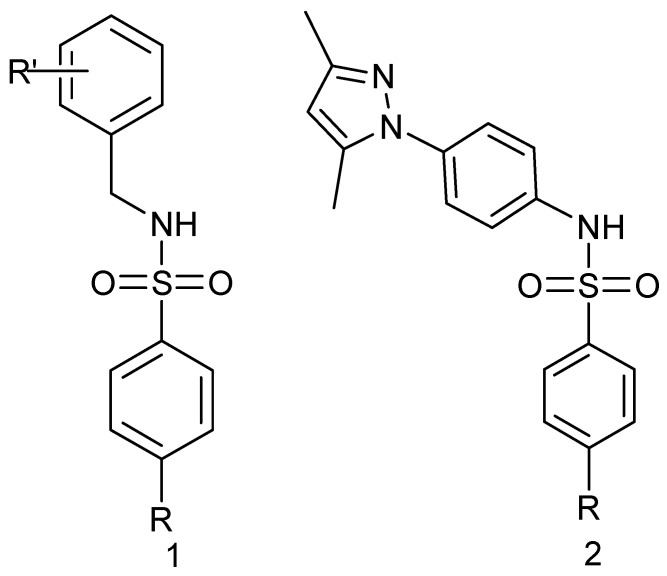
Structures of sulfonamides 1 and 2 prepared in this work (R = Me, NHAc; NO_2_; NH_2_), (R’ = 4 Cl; 4-OMe; 2,5-diOMe).

The *in-house* library was used to perform the VS by docking. MM–GBSA-binding free energy calculations were performed for the molecules obtained from the VS and compared with BIBR1532. Hit compounds that exhibited ∆G binding values (1A, 1B, 1C, 1E, 1G, 2B, and 2C, −70.75/−62.97 kcal/mol) lower than that obtained for BIBR1532 (−62.76 kcal/mol) were selected for synthesis and in vitro tests together with hit compounds retrieved using the MYSHAPE approach (1D, 1F, and 2A) (Figure 3).

### 2.2. Synthesis

For the preparation of sulfonamide compounds of type 1 and 2 (Figure 3), several procedures are present in the literature [54,55,56,57,58,59,60,61], and a few of them were also proposed recently in light of using more environmentally friendly green chemistry approaches [62,63,64,65,66].

**Scheme 1:**
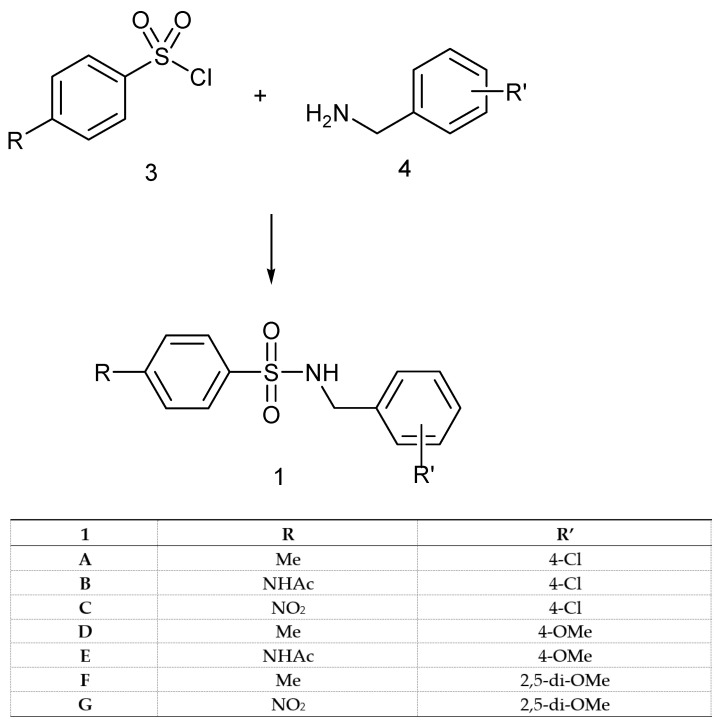
The general method for the synthesis of benzyl-sulfonamides of type 1.

**Scheme 2:**
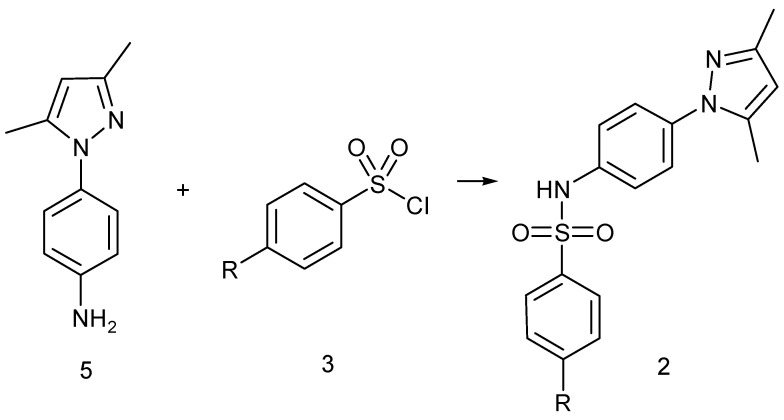
General method for the synthesis of sulfonamide 2, R = Me (2A); NO2 (2B); NH2 (2C).

Our approach to targeting derivatives of type 1 and 2 involved reactions of the sulfonyl chloride 3 and suitable benzylamines 4 or amine 5, according to Scheme 1 and Scheme 2. The benzylamines were commercially available, whereas the 1-(4-aminophenyl)-3,5-dimethylpyrazole (5) was prepared in two steps from acetylacetone and 4-nitrophenylhydrazine and the subsequent reduction of the nitro group with H_2_ and Pd/C, using standard literature methodology (see Section 4). 

In all cases, initially, the reactions were carried out under classical literature conditions, according to Methods X or Y (see Section 4) affording derivatives 1 in good to high isolated yields (64–97%) and slightly lower (49%) only in the case of 1E, probably because of the solubility problem of the starting material (Scheme 1). 

The aminophenyl-3,5-dimethylpyrazole 5 also reacted with sulfonylchloride 3 in anhydrous THF in the presence of an equimolar amount of triethylamine, affording sulfonamides 2A,B (Scheme 2).

The possibility of avoiding the use of dangerous/dry solvents was also considered. The pilot reaction of benzylamine and para-toluensulfonyl chloride, in Schotten–Baumann conditions under pH control with Na_2_CO_3_, as discussed in [67], was explored, but the result was the incomplete conversion to the desired sulfonamide. Recently, a new environmentally safe methodology was reported for the preparation of a wide range of aliphatic- and aryl-sulfonamides [68], and although the methodology was not extended to investigate the reactivity of benzylamines, we decided to explore whether this kind of amine resulted in a suitable substrate to prepare sulfonamides via this route. The green method afforded yields from good to quantitative, giving in some cases a similar efficient conversion of the reactants into products, if compared to Method X (Table 1). The major concern of this methodology is the competitive reactions leading to bis-sulfonylation which, however, in the case benzylamines were not observed, suggesting that this procedure has preferable results, especially when electron-withdrawing groups are present in both the reactants (as in the case of entry 1C).

Compound 2C was prepared by reduction of the corresponding nitro compound with H_2_/Pd in ethanol a quantitative yield. 

### 2.3. Cytotoxic Activity and Selectivity Index

To select the most promising cytotoxic agents, the newly synthesized compounds were initially evaluated on human colorectal carcinoma (HCsT-116) [69,70], human breast adenocarcinoma (MCF-7) [70,71], and human chronic myeloid leukemia (K-562) [71,72] cell lines that express the active telomerase.

As shown in Table 2, except for compound 2C, the antiproliferative efficacy of the compounds was modest and only evident at the highest micro-submillimolar concentrations. Moreover, compounds 1B, 1D, 1E, 1F, 1G, and 2B exhibited solubility issues that prevented the evaluation of their cytotoxicity. 

On the other hand, compound 2C revealed an interesting activity, and it was possible to calculate its IC_50_ (Table 3). Interestingly, this value on K-562 cells appeared 3.6-fold lower than that on HCT-116 and 4.2-fold lower than that on MCF-7 cells. Moreover, in the K-562 cell line tested, compound 2C showed an IC_50_ 6.8-fold lower than the reference compound BIBR1532, and an IC_50_ comparable to BIBR1532 in the MCF-7 cell line.

Selective cytotoxicity is a pivotal requirement for anticancer drugs. To determine the selectivity of compound 2C, its cytotoxicity against the cancer cell lines employed (i.e., HCT-116, MCF-7, and K-562) was compared with that against the non-cancerous, murine, embryonic, fibroblast cell line BALB/3-T3. As shown in Table 3, the IC_50_ of compound 2C on BALB/3-T3 cells was remarkably higher than those on the tumoral cell lines tested. Moreover, the calculated selectivity indexes (SIs) of compound 2C for HCT-116, MCF-7, and K-562 cells were 2.9, 2.5, and 9.8, respectively. Relevantly, these values are above the accepted threshold for antitumor drugs (SI = 2.0) [72,73].

Although selectivity for cancer cells cannot be easily derived from the comparison of toxicity parameters in different cell cultures, these data indicate that compound 2C shows preferential toxicity towards cancer cells. Along these lines, the potential use of compound 2C as a novel lead molecule for the development of more potent and selective antiproliferative agents can, therefore, be envisaged.

### 2.4. Docking of Compound 2C

**Figure 4:**
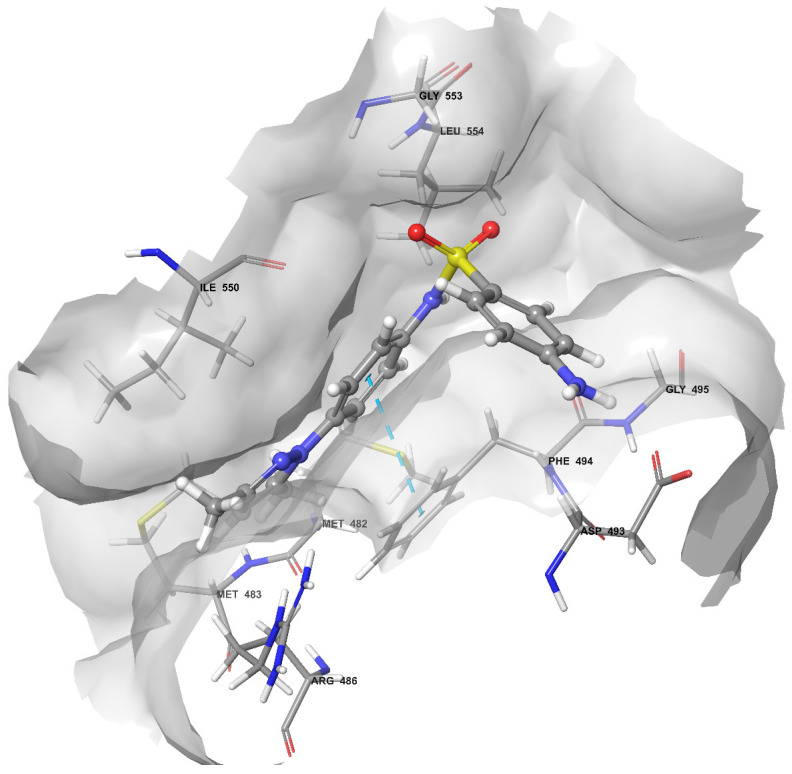
Binding mode of the best docking pose of 2C after the docking study. The light blue, dashed lines represent the pi–pi stacking. The displayed residues establish positive vdW contacts with the compound.

Analysis of the best docking pose of compound 2C showed a pi–pi stacking interaction between Phe494 and the central aromatic ring as observed in BIBR1532, where the same residue is involved in two H-aromatic bonds with the naphthyl ring (Figure 2). The same residues Phe494, Asp493, and Gly495 establish positive van der Waals (vdW) contacts with the aniline group of 2C. Other positive vdW contacts are formed by the sulfonamide moiety of 2C and Ile550, Gly553, and Leu554. The major part of positive vdW contacts are established by the dimethylpyrazolyl moiety with Met482, Met483, Arg486, Phe494, and Ile550 (Figure 4). 

### 2.5. Molecular Dynamics Simulation

The running of dynamics simulations of protein–ligand complexes over time could be considered a major step toward accuracy in computer-assisted drug design. In this study, an unbiased molecular dynamics simulation was performed to investigate the conformational stability and the time-dependent binding capability of 2C in the active site of the telomerase. Additionally, we tried to understand if the protein target undergoes conformational alteration after interacting with 2C. Therefore, starting from the previous docking, 100 ns of MD simulation was carried out. Various analyses, such root mean square deviation (RMSD), root mean square fluctuation (RMSF), and determination of the number and types of protein–ligand contacts, were performed to obtain a more detailed analysis of the 2C–target complex.

#### 2.5.1. Stability Analysis

**Figure 5:**
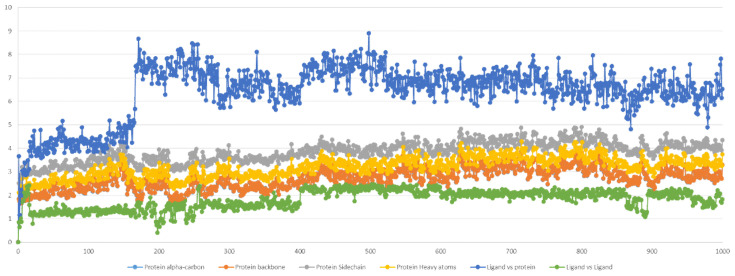
Compound 2C and protein RMSD during MD simulation (100 ns). The *x*-axis is expressed by the number of snapshots extracted, and 1 snapshot = 100 ps; the *y*-axis is expressed in Å.

The RMSD was selected as a criterion to evaluate the dynamic stability of the ligand-bound system [73]. The RMSD values of the protein’s atoms and ligand are reported in Figure 5. The system reached equilibrium quickly and fluctuated around the average RMSD value of <3 Å. The average value of ligand vs. protein RMSD of ~6.4 Å indicated a strong stability of 2C in the binding pocket compared to the ligand vs. ligand RMSD of ~1.81 Å.

#### 2.5.2. Residue Mobility and Protein–Ligand Contact Analyses

**Figure 6:**
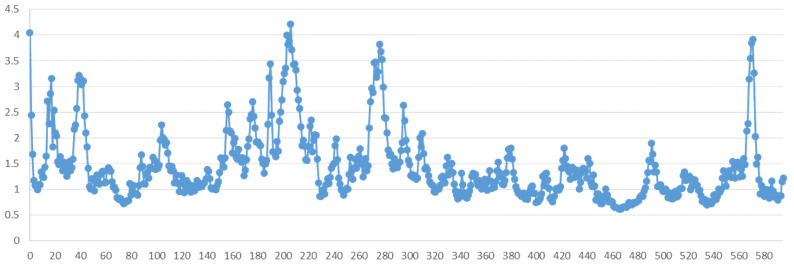
Protein RMSF during MD simulation (100 ns). The *x*-axis reports the number of the residues of TERT, and the *y*-axis is expressed in Å.

To examine the structural flexibility effect of 2C upon TERT per residue, the main chain average of the root mean square fluctuation (RMSF) of the complex was calculated for the entire 100 ns of simulation. The residue-wise fluctuation of the complexes was plotted and is presented in Figure 6. As reported, the RMSF plot was low for the identified residues involved in the interactions with 2C in the docking analysis.

**Figure 7:**
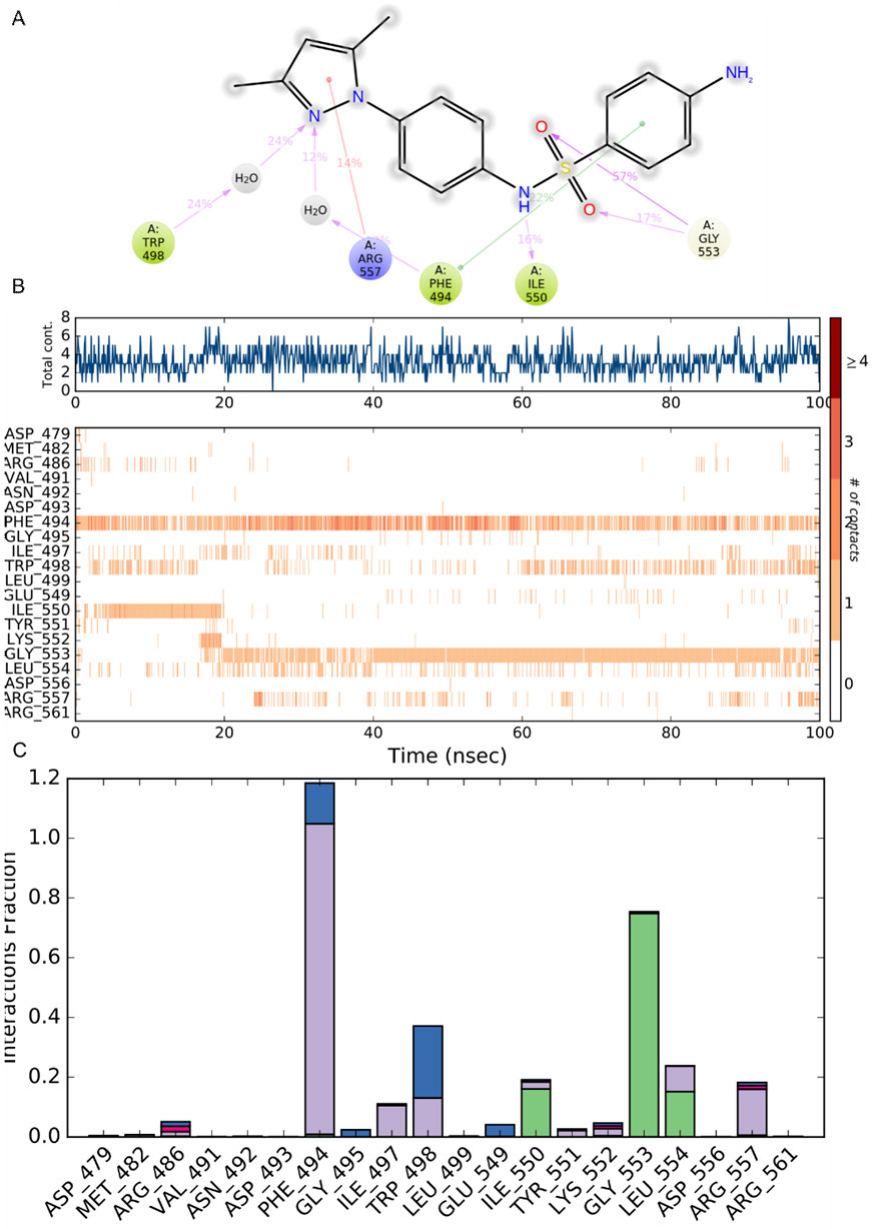
Protein–compound 2C contacts: (**A**) the interactions that occur for more than 10% of the simulation time are reported; (**B**) a timeline representation of the interactions and contacts (H-bonds, hydrophobic, ionic, and water bridges) during the simulation; (**C**) histogram of protein–compound 2C interactions fractions: hydrogen bonds (green), hydrophobic (purple), ionic (red), and water bridges (blue).

Evaluation of the protein interactions provides a measure of the interaction power between the ligands and the protein and can be categorized by type and summarized as represented in Figure 7. In Figure 7A, the interactions that occurred for more than 10% of the simulation time are reported. In Figure 7B, a timeline representation of the interactions and contacts (H-bonds, hydrophobic, ionic, and water bridges) summarize the total number of specific contacts the protein makes with the ligand throughout the simulation. The bottom panel of Figure 7B shows which residues interact with the ligand in each trajectory frame. Some residues make more than one specific contact with the ligand, and they are represented by a darker shade of orange, according to the scale to the right of the plot. Protein–ligand contacts are categorized into four types: hydrogen bonds, hydrophobic, ionic, and water bridges. The stacked bar charts are normalized throughout the trajectory: a value of 1.0 suggests that 100% of the simulation time, the specific interaction is maintained. 

The analysis of the trajectory evidenced that the pi-stacking interaction between the aniline ring of 2C and Phe494 occurred at 57%. This output evidences a shifting in the pi-stacking interaction with respect to the docking where the central phenyl ring was involved, but it clearly defines Phe494 as a key residue in the inhibition pattern. As also reported in Figure 7B,C, Phe494 established at least one contact for the entire duration of the simulation and, for a major part of the time, 2–3 contacts with the phenyl ring of 2C. Gly553 showed for 57% and 17% of the time two H-bonds interactions with the oxygen of the sulfonamide group emerging after ~18 ns of simulation. Interestingly, after 22 ns of the simulation a new in pi–cation interaction appeared for 14% of the time between Arg557 and the pyrazole ring, even though it was in a spotted fashion. During the first 20 ns (16% of the total time), the protein–ligand interaction stabilized with an H-bond between Ile550 and the NH of the sulfonamide group. It is interesting evidence of the role of a water molecule as a water bridge between Trp498 and the pyrazole ring. Other residues involved in the protein–ligand interaction but with a minor role were Arg486, Ile497, Trp498, Tyr551, Lys552, and Leu554.

#### 2.5.3. ADME Calculation for Compound 2C

**Figure 8:**
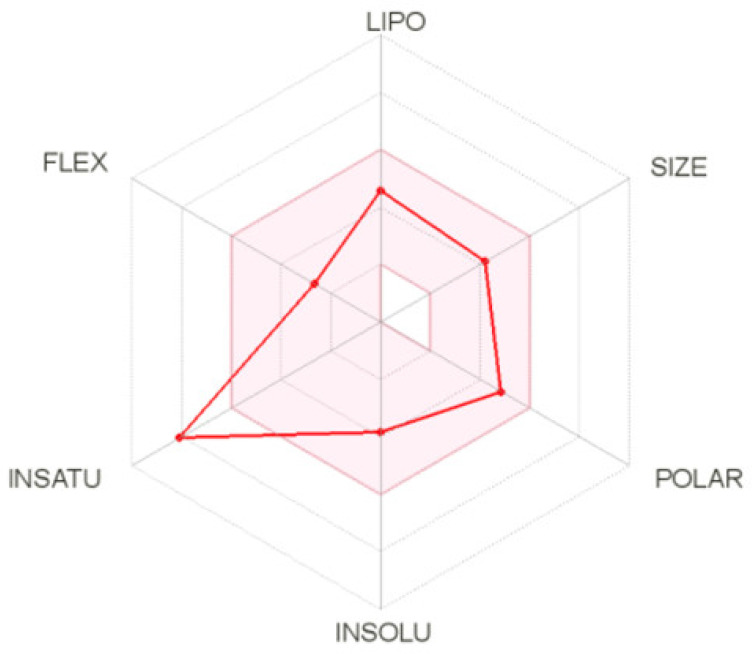
The bioavailability radar (the pink area exhibits the optimal range of a particular property) for compound 2C (LIPO = lipophilicity as in XLOGP3; SIZE = size as in molecular weight; POLAR = polarity as TPSA (topological polar surface area); INSOLU = insolubility in water by log S scale; INSATU = insaturation per fraction of carbons in the sp3 hybridization; FLEX = flexibility per rotatable bonds).

ADME calculation for compound 2C was performed using the SwissADME web tool [74]. In the hexagon drug-likeness graph (Figure 8), each vertex represents a parameter that defines a bioavailable drug. The pink regions represent the optimum range of the following six properties: lipophilicity = 2.53 (XLOGP3 between −0.7 and +5.0), size = 342.42 g/mol (MW between 150 and 500 g/mol), polarity = 98.39 Å^2^ (TPSA between 20 and 130 Å^2^), solubility (log S not higher than 6), saturation = 0.12 (fraction of carbons in the sp3 hybridization not less than 0.25), and flexibility (no more than nine rotatable bonds). It was found that compound 2C was slightly outside the pink area on one side due to the inconformity of its insaturation. The pharmacokinetics analysis showed that compound 2C is probably not a P-glycoprotein (P-gp) substrate and not BBB permeant. It has potentially good gastrointestinal (GI) absorption and ABS, up to nearly 70%. The %ABS, a very functional physiochemical variable, defines a drug’s transport properties. It was calculated according to the equation %ABS = 109 − (0.345 × TPSA) [75,76]. TPSA values below 98.39 Å^2^ characterize a significant permeability in the cellular plasma membrane. It has a bioavailability score of 0.55, which means good pharmacokinetic properties according to the Rule of 5 by Lipinski [77].

## 3. Discussion

In the past decade, there has been tangible progress in the definition of the role of telomerase in tumor progression. Several studies evidenced telomerase as a promising target for developing cancer therapeutics. The main reason is due to the overexpression of telomerase in cancer cells (85–90%) compared with normal cells where it is almost unexpressed. Despite the fact that efforts have allowed for the identification of several small molecules, oligonucleotides, natural products, and immunotherapeutics, no telomerase-based cancer drugs have yet been approved by the FDA due to the long lag time between administration of the drug and clinical response. For these reasons, the identification and then the optimization of new small molecules, such as telomerase inhibitors, remain a crucial point that deserves to be further explored. A structure-based drug design approach could be used to design potential inhibitors of the telomerase active site. Exploiting the experimental structure of TERT showing a known inhibitor co-crystallized, we developed a combined structure-based strategy to screen an *in-house* library of 126 arylsulfonamide derivatives. The combined computational strategy was performed using the MYSHAPE approach, first, to identify hit compounds. Successively, docking and calculation of the binding free energy using the MM-GBSA method identified other hits. These approaches guided the selection of the most promising compounds for synthesis and in vitro tests. 

As described in Section 2.2., the compounds were initially prepared using classical methodologies. To explore the possibility of avoiding using dangerous and/or dry solvents, a reported green procedure was successfully utilized in the case of derivatives listed in Table 1, affording yields from good to quantitative for a major part of the compounds.

With the aim of identifying the most promising cytotoxic agents, the selected hits, newly synthesized, were evaluated on three cancer lines: human colorectal carcinoma (HCT-116), human breast adenocarcinoma (MCF-7), and human chronic myeloid leukemia (K-562) cell lines, considering the known telomerase inhibitor BIBR1532 as the reference compound. One of the major problems faced during the in vitro test was the poor solubility for some of the tested compounds, which prevented the evaluation of the cytotoxicity and gave results in micro-submillimolar concentrations. In general, the pyrazole derivatives (2A, 2B, and 2C) seems to show a higher activity on the tested cell lines, probably even due to the major solubility 

One of the selected hits (2C) revealed an interesting IC_50_ in all three cell lines. In particular, the IC_50_ results were comparable with BIBR1532 for the HCT-116 cell line and the MCF7 cell line, but several times lower in the K-562 cell lines. These findings were confirmed by the evidence of the selective cytotoxicity against the non-cancerous, murine, embryonic, fibroblast cell line BALB/3-T3. The IC_50_ of compound 2C on BALB/3-T3 cells was remarkably higher than those on the tumoral cell lines tested. Another interesting concern regarding the data was the absence of the solubility issue encountered during the in vitro test for the compound 2C in addition to the calculated ADME values that seemed to exclude a binding with the P-gp, an important parameter for the insurgence of resistance issues. In the end, compound 2C showed a good calculated pharmacokinetic profile. Further, the MD simulation of compound 2C at the telomerase active site showed good stability and evidenced Phe494 as the key residue also in BIBR1532. But the more interesting evidence of the simulation regarded that each moiety of compound 2C was involved in key interactions with some residues of the active site: the pyrazole moiety in a cation–pi-stacking interaction with Arg557, the two phenyl rings in the pi-stacking interaction with Phe494, and the sulfonamide moiety in the H-bond interaction with Ile550 and Gly553. Compound 2C as arylsulfonamide derivative is an interesting hit compound that deserves, for the reasons stated above, further investigation in terms of the optimization of its structure to obtain more active telomerase inhibitors.

## 4. Materials and Methods

### 4.1. Library and Protein Preparation 

The library used for this experimental work comprised a series of 126 sulfonamide derivates. The sulfonamide and BIBR1532 structures were built using the builder panel in Maestro, and ligand preparation was carried out by LigPrep (Schrödinger, LLC, New York, NY, USA, 2021). The force field adopted was OPLS4 [78] and Epik 5.5 (Schrödinger, 2021-1) was selected as an ionization tool at pH 7.2 ± 0.2. Tautomer generation was unflagged, and the maximum number of conformers generated was set at 32. For the purpose of this study, the 2.30 Å resolution crystal structure of *Tribolium castaneum* telomerase in complex with the highly specific inhibitor BIBR1532 (PDB ID: 5CQG) [21] was used. The structure was optimized using the Protein Preparation Wizard in Maestro (Schrödinger, 2021-1) adding bond orders and hydrogen atoms to the crystal structure using the OPLS4 force field. The remaining settings are reported in [79].

### 4.2. Docking Studies 

The library was submitted to a docking study using Glide v9.0 [80] in standard precision (SP) with the OPLS4 force field. The grid box was built considering the BIBR1532 as the centroid of the grid. The study was performed using no constraints. Van der Waals radii were set at 0.8, and the partial cutoff was 0.15 with flexible ligand sampling. Bias sampling torsion penalization for amides with non-planar conformation and Epik state penalties were added to the docking score.

### 4.3. MYSHAPE Approach

MYSHAPE [51] is an approach in which the pharmacophore model is built using only the common pharmacophore feature patterns that the ligands exhibit during MD simulations. The evaluation of the ligand–protein interaction patterns during the MD simulation was investigated using the data recorded employing the MD simulation (exactly 1001 frames for each simulation). The MYSHAPE model was used to distinguish this type of model from the “default” pharmacophore models that were generated using the crystal structure of the ligand–protein complex. For the newly added pharmacophore features, the tolerance radius was increased by 0.15 Å to compensate for small deviations in the 3D coordinates. The computational details are reported in previously published papers [51,52]

### 4.4. MM-GBSA Free Energy Calculations

The output of docking was used to calculate the ΔG values of the BIBR1532 and molecules using MM-GBSA (molecular mechanics generalized-born surface area) (Prime, Schrödinger, LLC, New York, NY, USA, 2021). Protein–ligand binding free energy using MM-GBSA was calculated as the difference between the energy of the bound complex and the energy of the unbound protein and ligand. The entropy term −TΔS was not calculated to reduce computational time as previously reported [79,81,82,83]. The VSGB solvation model was chosen using OPLS4 force field with the minimized sampling method.

### 4.5. Molecular Dynamic Simulations

A 100 ns MD simulation was carried out using a Desmond 6.5 (Desmond Molecular Dynamics System, D. E. Shaw Research, New York, NY, USA, 2021) using the OPLS4 force field for the complex TERT/2C. The complex was solvated in a cubic box using the TIP3P. Ions were added to neutralize charges. The systems were minimized and equilibrated at a temperature of 303.15 K and a pressure of 1.013 bar. The system was simulated as an NPT ensemble; a Nose–Hover thermostat and Martyna–Tobia–Klein barostat were used. The integration time step was chosen to be 2 fs. To keep the hydrogen-heavy atom bonds rigid, the SHAKE algorithm was used. A 9 Å cutoff radius was set for the short-range Coulomb interactions, and smooth particle mesh Ewald was used for the long-range interactions. The detection ranges for energy were 1.2 ps, and 5.0 ps for the trajectory. 

### 4.6. ADME Prediction

Drug-likeness, physicochemical properties, lipophilicity, solubility, and pharmacokinetics properties were analyzed by the SwissADME web tool (http://www.swissadme.ch/index.php (accessed on 30 November 2021)). SwissADME is a new comprehensive tool run by the Swiss Institute of Bioinformatics (SIB) enabling estimation of ADME (absorption, distribution, metabolism, and excretion) parameters of drug candidates. 2D structural models of the compound were drawn in the molecular sketcher into the ChemAxon’s Marvin JS window and transferred to the SMILES (simplified molecular-input line-entry system) format to predict suitable properties [74].

### 4.7. Chemistry

All melting points were taken on a Büchi melting point M-560 apparatus and were uncorrected. IR spectra were recorded in Bromoform with a JASCO FT-IR spectrophotometer. 1H and 13C NMR spectra were measured at 200 and 50.0 MHz, respectively, in DMSO-d_6_ solution and TMS as an internal standard, using a Bruker Avance II Series 200 MHz spectrometer or at 300 and 75 MHz (APT) with a Bruker AC-E spectrometer. Column chromatography was performed with Merck silica gel (230–400 mesh ASTM). Elemental analyses (C, H, and N) were within ±0.4% of theoretical values. The substituted benzylamine derivatives were commercially available and were used without further purification. For all the compounds already cited in the literature, the IR and NMR spectra results were identical to those reported (1A–E). The 1H and 13C NMR spectra of new synthesized compounds (1F,G and 2A–C) are reported in the Supplementary Materials.

#### 4.7.1. General Methods for the Preparation of *N*-(R’-Benzyl)-4-R-benzenesulfonamides of Type 1

Method X: To a stirred solution of amino derivative (1.5 mmol) in anhydrous THF (20 mL), an equimolar amount of triethylamine (0.21 mL) and the suitable sulfonylchloride were added. The reaction mixture was stirred for the appropriate time-lapse until the disappearance of the starting amine (TLC monitoring). The ammonium salt was collected and taken up with 20 mL H_2_O. The aqueous solution was extracted using DCM. The organic layers were combined with the organic mother liquor, dried over Na_2_SO_4_, and concentrated under reduced pressure to yield the desired sulfonamide of type 1.

Method Y: The amino derivative (5 mmol) was dissolved in dry pyridine, and the suitable sulfonylchloride (5 mmol) was added. The reaction mixture was stirred for the appropriate time-lapse until the disappearance of starting amine (TLC monitoring). The mixture was poured onto ice/water and the solid precipitate was collected by filtration, air-dried, and recrystallized from ethanol.

##### *N*-(4-Chlorobenzyl)-4-methylbenzenesulfonamide (1A) 

This compound was prepared according to Method X. The reaction mixture was stirred for 24 h at room temperature. Yield 70%. Mp 98 °C ([84] 107–108 °C; [85] 106.1 °C). 1H NMR ppm: 2.43 (s, 3H, Me), 4.06 (d, J = 6.4 Hz, 2H, CH_2_), 5.18 (t, J = 6.0 Hz, 1H, NH), 7.11 (d, J = 8.4 Hz, 2H, Ar-H), 7.20 (d, J = 8.4 Hz, 2H, Ar-H), 7.27 (d, J = 8.0 Hz, 2H, Ar-H), 7.70 (d, J = 8.4 Hz, 2H, Ar-H). 13C NMR ppm: 21.6, 46.6, 127.2, 128.8, 129.3, 129.9, 133.7, 135.1, 136.9, and 143.8.

##### *N*-(4-Chlorobenzyl)-4-acetylaminobenzenesulfonamide (1B) 

This compound was prepared according to Method X. The reaction mixture was stirred for 24 h at room temperature and under reflux for an additional 4 h. Yield 64%. Mp 152°C [56] 172–174 °C). 1H NMR ppm: 2.10 (s, 3H, Me), 3.08 (bs, 1H, NH), 3.96 (s, 2H, CH_2_), 7.30–8.11 (m, 8H, Ar-H), 10.42 (bs, 1H, NH). 13C NMR ppm: 24.1, 45.5, 118.6, 127.6, 128.5, 129.4, 131.6, 134.1, 136.9, 142.5, and 169.0.

##### *N*-(4-Chlorobenzyl)-4-nitrobenzenesulfonamide (1C)

This compound was prepared according to Method X. The reaction mixture was stirred under reflux for 4.5 h. Yield 78%. Mp 174–176 °C ([86] 168–170 °C). 1H NMR ppm: 3.80 (d, J = 6.0 Hz, 2H, CH2), 6.61 (d, J = 8.0 Hz, 2H, Ar-H), 6.85 (d, J = 8.0 Hz, 2H, Ar-H), 7.15 (d, J = 8.0 Hz, 2H, Ar-H), 7.45 (d, J = 8.0 Hz, 2H, Ar-H), 7.56 (t, J = 6.0 Hz, 1H, NH). 13C NMR ppm: 45.5, 112.6, 113.5, 125.5, 128.4, 128.9, 129.8, 152.4, and 158.3.

##### *N*-(4-Methoxybenzyl)-4-methylbenzenesulfonamide (1D)

This compound was prepared according to Method X. The reaction mixture was stirred for 3 h at room temperature. Yield 71%. Mp 122–123 °C ([87] 114–117 °C; [88] 122–123 °C from EtOAc). 1H NMR (300 MHz, DMSO) ppm: 2.38 (s, 3H, Me), 3.72 (s, 3H, Me), 3.89 (s, 2H, CH_2_), 6.85 (d, J = 8.4 Hz, 2H, Ar-H), 7.17 (d, J = 8.4 Hz, 2H, Ar-H), 7.38 (d, J = 8.0 Hz, 2H, Ar-H), 7.71 (d, J = 8.4 Hz, 2H, Ar-H), 8.03 (bs, 1H, NH). 13C NMR ppm: 20.9, 45.8, 55.0, 113.6, 126.5, 128.9, 129.4, 129.5, 137.8, 142.5, and 158.4.

##### *N*-(4-Methoxybenzyl)-4-acetylaminobenzenesulfonamide (1E) 

This compound was prepared according to Method Y. The reaction mixture was stirred for 1 h at room temperature and under reflux for an additional 2 h. Yield 49%. Mp 192–193 °C ([87] 164–167 °C). 1H NMR ppm: 1.84 (s, 2H, CH_2_), 2.12 (s, 3H, Me), 4.46 (sa, 1H, NH), 7.74 (d, J = 8.5 Hz, 2H), 7.79 (d, J = 8.5 Hz, 2H), 9.41 (sa, 1H, NH). 13C NMR ppm: 24.2, 45.6, 55.2, 113.6, 118.6, 127.6, 129.1, 129.5, 134.0, 142.9, 158.5, and 169.4

##### *N*-(2,5-Dimethoxybenzyl)-4-methylbenzenesulfonamide (1F)

This compound was prepared according to Method Y. The reaction mixture was stirred for 10 h at room temperature. Yield 80%. Mp 110–111 °C. IR ν: 3268 (NH) cm^−1^. 1H-NMR ppm: 2.37 (3H, s, Me), 3.65 (6H, s, Me), 3.91 (2H, t, J = 3.9 Hz, CH_2_), 6.77–6.83 (3H, m, ArH), 7.10 (2H, d, J = 8.5 Hz), 7.36 (2H, d, J = 7.6 Hz), 7.68 (2H, d, J = 7.6 Hz), 7.89 (1H, t, J = 3.9 Hz, NH). 13C NMR ppm: 21.4, 41.3, 55.8, 56.1, 111.8, 113.0, 115.0, 126.7, 127.0, 130.0, 138.2, 143.0, 150.9, and 153.4.

##### *N*-(2,5-Dimethoxybenzyl)-4-nitrobenzenesulfonamide (1G)

This compound was prepared according to Method X. The reaction mixture was stirred for 24 h at room temperature. Yield 97%. Mp 129–132°C. ir ν: 3268 (NH), 1523 and 1348 (NO_2_) cm^−1^. 1H NMR ppm: 3.35 (s, 3H, Me), 3.63 (s, 3H, Me), 4.03 (s, 2H, CH_2_), 6.65–6.86 (m, 3H, Ar), 7.96 (2H, d, J = 8.8 Hz), 8.34 (2H, d, J = 8.8 Hz). 13C NMR ppm: 46.0, 55.7, 56.1, 111.8, 113.2, 115.5, 124.7, 125.9, 128.4, 147.0, 149.8, 151.0, and 153.2.

#### 4.7.2. Preparation of 1-(4-Aminophenyl)-3,5-dimethylpyrazole

A solution of acetylacetone (10 mmol) and 4-nitrophenylhydrazine (10 mmol) in acetic acid was heated under reflux for 9 h. The reaction mixture was cooled to room temperature and poured onto ice/water to give a solid precipitate. Purification by column chromatography (eluant DCM/EtOAc 4:1) gave the nitro compound. Yield 80%, mp 100 °C ([89] 102 °C from EtOH). 1-(4-Nitrophenyl)-3,5-dimethylpyrazole (0.56 mg) dissolved in EtOH (20 mL) Pd/C was added, and the mixture was reduced overnight at room temperature in an H2 atmosphere (50 psi) in a Parr apparatus. The catalyst was filtered off, and the solution was evaporated under reduced pressure. The amino derivatives were isolated as white crystals. Yield 100%. Mp 84 °C ([90] 82–84 °C from benzene).

#### 4.7.3. General Method for the Preparation of *N*-[4-(3,5-Dimethyl-1H-pyrazol-1-yl)phenyl]-4-R-benzenesulfonamide of Type 2

To a stirred solution of 1-(4-aminophenyl)-3,5-dimethylpyrazole (1.5 mmol) in anhydrous THF (20 mL), an equimolar amount of triethylamine (0.21 mL) and the suitable sulfonylchloride were added. The reaction mixture was stirred for the appropriate time-lapse until the disappearance of the starting amine (TLC monitoring). The ammonium salt was collected and taken up with 20 mL H_2_O. The aqueous solution was extracted using DCM. The organic layers were combined with the organic mother liquor, dried over Na_2_SO_4_, and concentrated under reduced pressure to yield the desired sulfonamide. 

##### *N*-[4-(3,5-Dimethylpyrazol-1-yl)phenyl]-4-methylbenzenesulfonamide (2A)

The reaction mixture was stirred for 30 h at room temperature. Yield 96%. Mp 167 °C. ir ν: 3392 (NH) cm^−1^. 1H NMR ppm: 2.09 (s, 3H, Me), 2.13 (s, 3H, Me), 2.28 (s, 3H, Me), 6.00 (s, 1H, pyrazole-CH), 7.20 (dd, 4H, ArH), 7.27 (d, 2H, ArH), 7.64 (d, 2H, ArH), 10.41 (s, 1H, NH). 13C NMR ppm: 12.3, 13.4, 21.3, 107.5, 120.7, 125.5, 125.8, 127.2, 128.8, 130.3, 136.7, 134.0, 144.2, and 148.6.

##### *N*-[4-(3,5-Dimethylpyrazol-1-yl)phenyl]-4-nitrobenzenesulfonamide (2B) 

The reaction mixture was stirred for 3 h at room temperature. The solid residue was recrystallized from ethanol. Yield 57%. Mp 219 °C. ir ν: 3392 (NH), 1532, and 1348 (NO_2_) cm^−1^. 1H NMR ppm: 2.12 (s, 3H, Me), 2.21 (s, 3H, Me), 6.02 (s, 1H, pyrazole-CH), 7.27 (dd, 4H, ArH), 8.19 (dd, 4H, ArH), 10.81 (s, 1H, NH). 13C NMR ppm: 12.0, 13.2, 107.0, 120.9, 124.7, 125.0, 128.3, 132.2, 135.5, 136.2, 139.0, 147.7, and 149.9.

##### Preparation of *N*-[4-(3,5-Dimethylpyrazol-1-yl)phenyl]-4-aminobenzenesulfonamide (2C) 

To the nitro derivative, 2B dissolved was in EtOH (20 mL), Pd/C was added, and the mixture was reduced overnight at room temperature in an H_2_ atmosphere (50 psi) in a Parr apparatus. The catalyst was filtered off, and the solution was evaporated under reduced pressure. The amino derivative was isolated as white crystals. Yield 100%. Mp 106–110 °C. ir ν: 3482 and 3380 (NH_2_), 3234 (NH) cm^−1^. 1H NMR ppm: 1.92 (s, 3H, Me), 2.33 (s, 3H, Me), 6.00 (s, 1H, pyrazole-CH), 7.30–7.35 (m, 4H, Ar-H), 7.42–7.46 (m, 4H, Ar-H), 13C NMR ppm: 12.0, 13.2, 112.6, 119.5, 122.6, 124.8, 125.0, 128.3, 130.3, and 152.9.

### 4.8. Cell Proliferation Assay

#### Cell Culturing and MTT Assay

Unless stated otherwise, all reagents were from Merck (Milan, Italy) and of the highest purity grade commercially available. All synthesized compounds were dissolved in dimethyl sulfoxide (DMSO) and then diluted in a culture medium so that the effective DMSO concentration did not exceed 0.1% (*v*/*v*). HCT-116, MCF-7, K-562, and BALB/3-T3 cell lines were purchased from the American Type Culture Collection, Rockville, MD, USA. Except for BALB/3-T3 cells, which were grown in DMEM, all other cells were cultured in the RPMI-1640 medium. Both DMEM and RPMI-1640 were supplemented with L-glutamine (2 mM), 10% fetal bovine serum (FBS), penicillin (100 U/mL), streptomycin (100 μg/mL), and gentamicin (5 μg/mL). Cells were maintained in the log phase by seeding them twice a week at a density of 3 × 10^5^ cells/mL, in humidified 5% CO_2_ atmosphere at 37 °C. 

The cytotoxic activity of the synthesized compounds against all the cell lines employed was determined by the MTT colorimetric assay as previously reported [91]. The assay is based on the reduction of 3-(4,5-dimethyl-2-thiazolyl)bromide-2,5-diphenyl-2-*H*-tetrazolium to purple formazan by the mitochondrial dehydrogenases of living cells. Briefly, cells at the passage that did not exceed the number 20, were seeded into 96-well plates (Corning, New York, NY, USA) at a density of 2.0 × 10^4^ cells/cm^2^, incubated overnight, and then treated with either the compounds or the vehicle (control) for 24 h. Afterward, the medium was carefully removed, and 200 μL of 5 mg/mL MTT was added. The supernatant was discarded after a 2 h of incubation at 37 °C, and the formazan blue formed dissolved in DMSO. The absorbance at 565 nm of the formazan product was measured using a microplate reader (LTek, INNO, Seongnam, South Korea), and the value of control cells was taken as 100% of viability. Each experiment was repeated three times in triplicate to obtain the mean values. No differences were found between cells treated with DMSO 0.1% and untreated cells in terms of cell number and viability. 

The growth inhibition activity of the tested compounds was defined as the IC_50_ value that represents the concentration of the compound that inhibits 50% of cell viability. IC_50_ values were calculated using the dose–response inhibition model in Prism 8 (GraphPad Software, San Diego, CA, USA). The SI, a measure of the therapeutic potential of the tested compound, was calculated by dividing the IC_50_ for normal BALB/3-T3 cells by the IC_50_ for HCT-116, MCF-7, and K-562 cancer cells.

Two hundred milliliters (6.0 × 104 cells/cm^2^) of a K-562 cell suspension were plated in each well of a 96-well plate and treated with different concentrations of compounds. An equal volume of DMSO was added to the control well, and the cells were cultured for an additional 24 h; then, 20 μL MTT (5 mg/mL) in the growth medium was added per well, and the plates were incubated at 37 °C for 4 h as reported in [91] with some modifications. Plates were then centrifuged at 400× *g* for 10 min. Supernatants were removed from the wells, and the reduced MTT dye in each well was solubilized in 200 μL DMSO. Absorbance was measured in a microplate reader (LTek, INNO, Seongnam, Korea), and the value of the control cells was taken as 100% of viability.

## Figures and Tables

**Table 1:** Comparison of the yields obtained using classical and green chemistry methods.

1	R	R’	Method X	No Solvent *
**C**	NO_2_	4-Cl	78	100
**D**	Me	4-OMe	100	60
**E**	NHAc	4-OMe	80	70

* According to [68], the amine (2 mmol) and anhydrous NaHCO_3_ (approximately 1 g) were ground together into a fine powder, and arylsulfonyl chloride (2 mmol) was added under vigorous stirring at room temperature. The progress of the reaction was monitored by TLC until the conversion of the amine was completed.

**Table 2:** Cytotoxicity of the synthesized compounds on human tumor cell lines HCT-116, MCF-7, and K-562. Cells were treated for 24 h in the absence or the presence of the compound tested at the indicated concentration, and cell viability was assessed by an MTT test as reported in Section 4. Values are expressed as the mean ± SD of three separate experiments conducted in triplicate. * Sol: solubility issue.

Compound				% Inhibition Cell Proliferation											
		HCT-116			MCF-7								K-562		
				Compound Concentration (µm)											
	10	75	150	300	400	10	75	150	300	400	10	75	150	300	400
**1A**	0	0	19 ± 2	29 ± 3	40 ± 2	0	0	0	12 ± 1	13 ± 1	0	0	0	33 ± 2	38 ± 2
**1B**	0	5 ± 0.2	23 ± 2	* Sol	* Sol	0	15 ± 1	21 ± 2	* Sol	* Sol	0	5 ± 0.3	21 ± 1	* Sol	* Sol
**1C**	0	0	2 ± 0.1	8 ± 0.2	15 ± 2	0	5 ± 0.1	15 ± 1	19 ± 1	30 ± 2	0	12 ± 1	13 ± 1	16 ± 1	25 ± 1
**1D**	0	0	0	* Sol	* Sol	0	0	0	* Sol	* Sol	0	0	0	* Sol	* Sol
**1E**	0	5 ± 0.1	* Sol	* Sol	* Sol	0	20 ± 2	* Sol	* Sol	* Sol	0	16 ± 1	* Sol	* Sol	* Sol
**1F**	0	0	6 ± 0.3	* Sol	* Sol	0	0	0	* Sol	* Sol	0	0	0	* Sol	* Sol
**1G**	0	5 ± 0.2	28 ± 2	* Sol	* Sol	0	12 ± 1	35 ± 2	* Sol	* Sol	0	2 ± 0.1	37 ± 3	* Sol	* Sol
**2A**	0	5 ± 0.1	30 ± 1	44 ± 2	55 ± 3	0	0	0	0	0	0	13 ± 1	42 ± 2	55 ± 3	60 ± 3
**2B**	0	0	37 ± 2	* Sol	* Sol	0	17 ± 1	34 ± 2	* Sol	* Sol	0	19 ± 1	23 ± 2	* Sol	* Sol
**2C**	15 ± 2	31 ± 2	56 ± 2	75 ± 2	91 ± 4	10 ± 1	19 ± 3	59 ± 2	76 ± 2	85 ± 2	35 ± 2	70 ± 3	76 ± 1	80 ± 3	85 ± 2
**BIBR-1532**	25 ± 2	47 ± 3	56 ± 3	89 ± 2	95 ± 5	12 ± 2	23 ± 2	57 ± 3	74 ± 2	91 ± 3	10 ± 2	19 ± 3	32 ± 1	62 ± 3	79 ± 2

**Table 3:** IC_50_ of 2C against HCT-116, MCF-7, K-562, and BALB/3-T3 cell lines. Values were calculated by plotting the percentage viability versus concentration on a logarithmic graph and are expressed as the mean ± SD of three separate experiments conducted in triplicate.

Compound	IC_50_ (µm)			
	HCT-116	MCF-7	K-562	BALB/3-T3
**BIBR-1532**	70 ± 4	120 ± 8	208 ± 11	N/A
**2C**	109 ± 8	127 ± 9	33 ± 4	324 ± 15

## Data Availability

The data presented in this study are available in article and Supplementary Material.

## Supplementary Materials

The following supporting information can be downloaded at: www.mdpi.com/article/10.3390/ph15010082/s1, 1H and 13C NMR spectra of new derivatives synthetized and tested in this work.
