# Supplementary material for: In Silico Design, Synthesis, and Biological Evaluation of Anticancer Arylsulfonamide Endowed with Anti-Telomerase Activity
Source: Pharmaceuticals (Basel). 2022 Jan 10;15(1):82. doi: 10.3390/ph15010082 (PMC8778141; doi:10.3390/ph15010082)

In Silico Design, Synthesis and Biological Evaluation of Anticancer Arylsulfonamide Endowed with Anti-Telomerase Activity

Giulia Culletta [1,2], Mario Allegra [2], Anna Maria Almerico [2], Ignazio Restivo [2] and Marco Tutone [2,*]

[1] Dipartimento di Scienze Chimiche, Biologiche, Farmaceutiche e Ambientali, Università di Messina, 98166 Messina, Italy; giulia.culletta@unime.it

[2] Dipartimento di Scienze e Tecnologie Biologiche Chimiche e Farmaceutiche, Università degli Studi di Palermo, 90123 Palermo, Italy; mario.allegra@unipa.it (M.A.); annamaria.almerico@unipa.it (A.M.A.); ignazio.restivo@unipa.it (I.R.)

* Correspondence: marco.tutone@unipa.it

1H and 13C NMR spectra of new derivatives synthetized and tested in this work

NMR spectra of derivative N-(2,5-Dimethoxybenzyl)-4-methylbenzenesulfonamide (1F)

1H

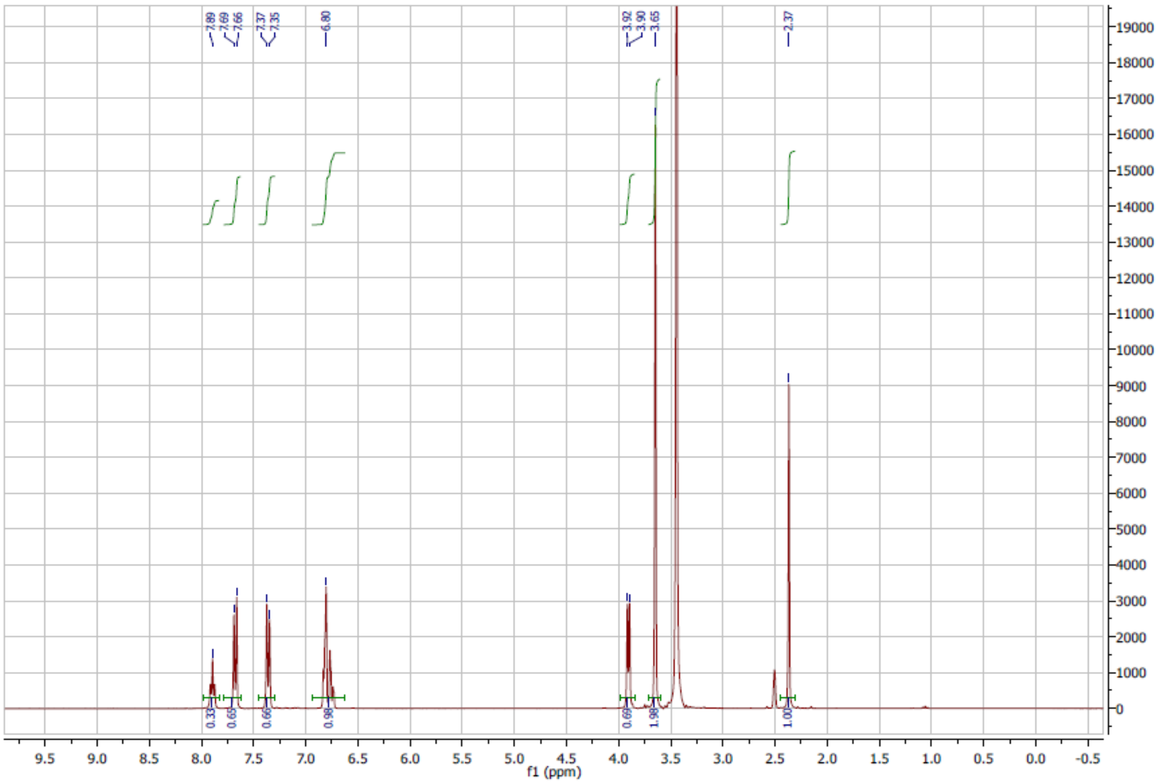

13C

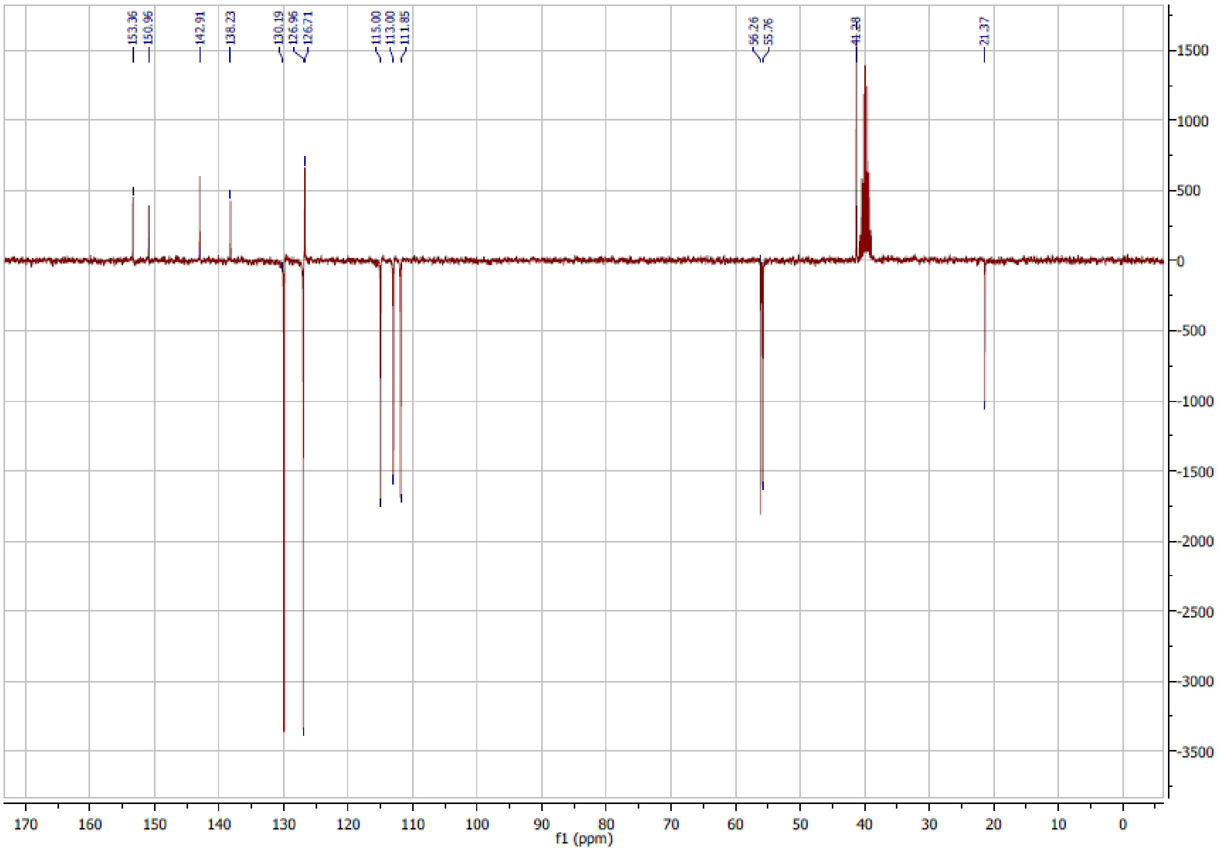

NMR spectra of derivative N-(2,5-Dimethoxybenzyl)-4-nitrobenzenesulfonamide (1G)

1H

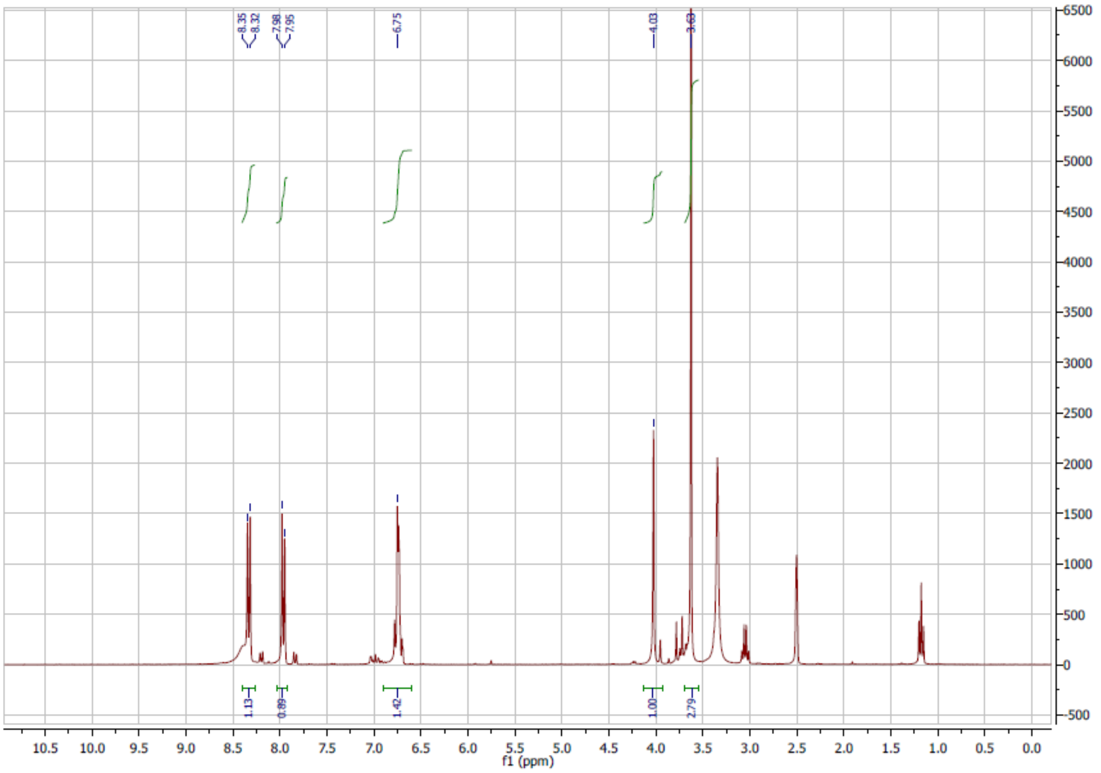

13C

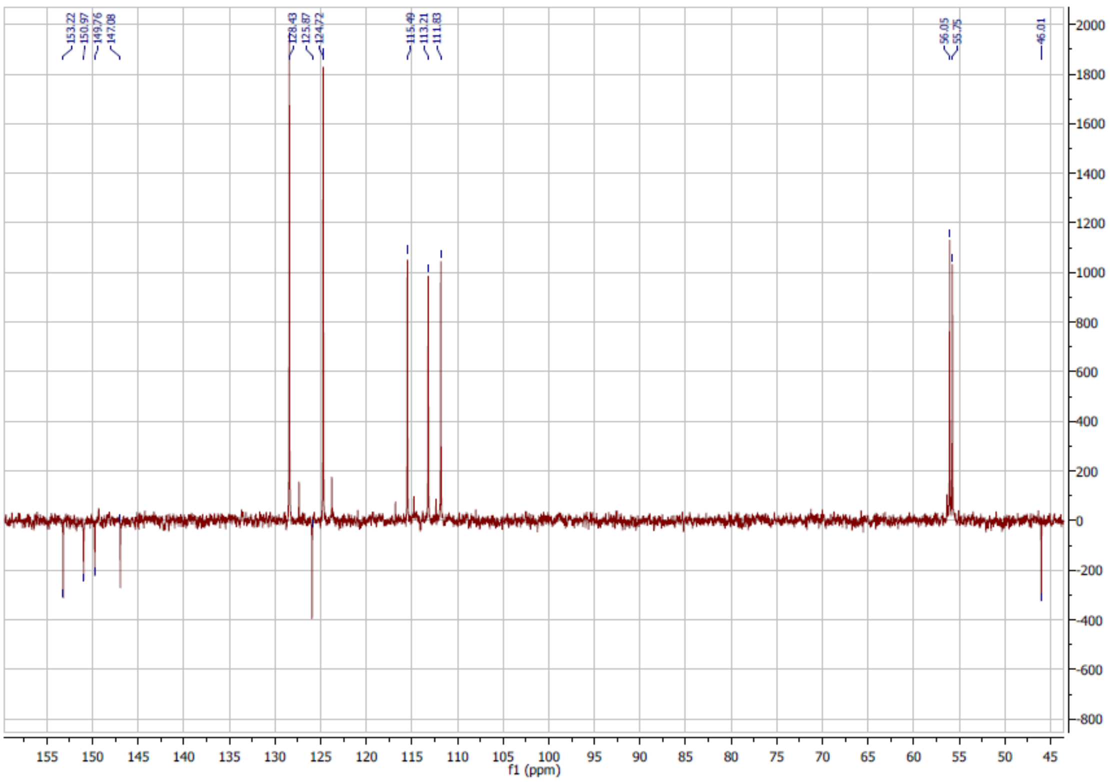

NMR spectra of derivative N-[4-(3,5-dimethylpyrazol-1-yl)phenyl]-4-methylbenzenesulfonamide (2A)

1H

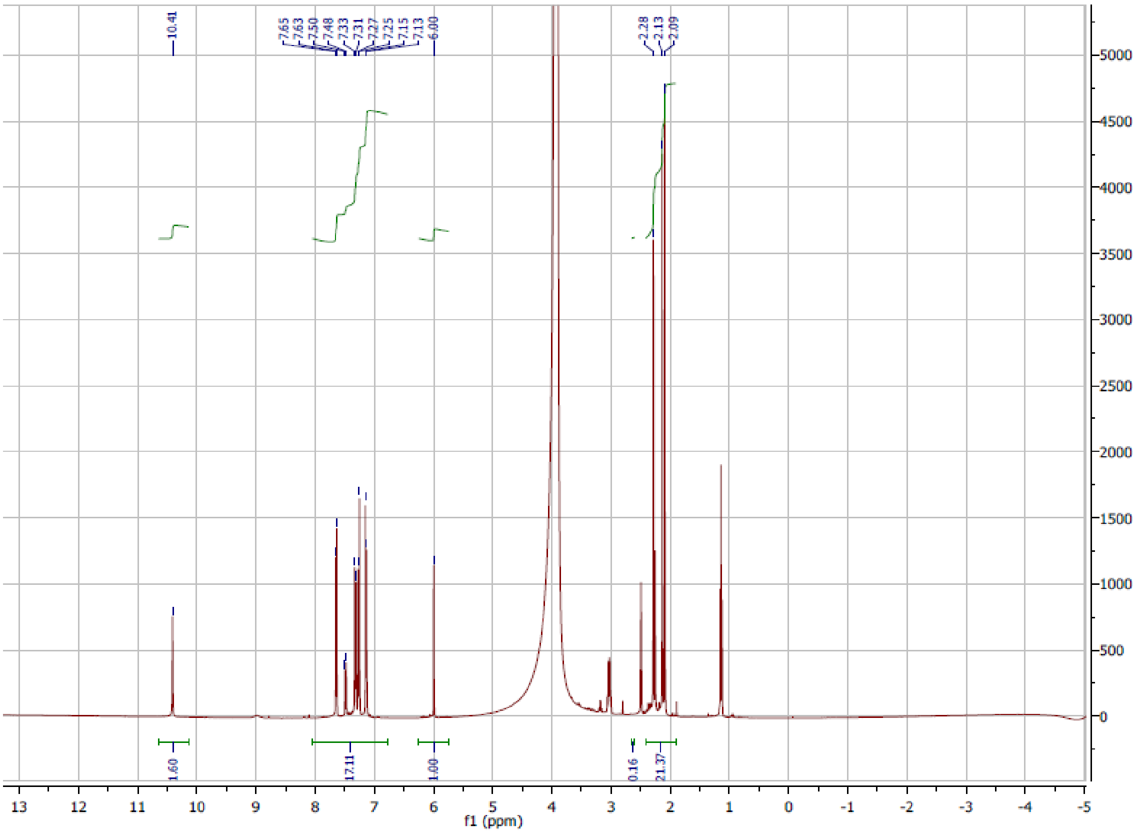

13C

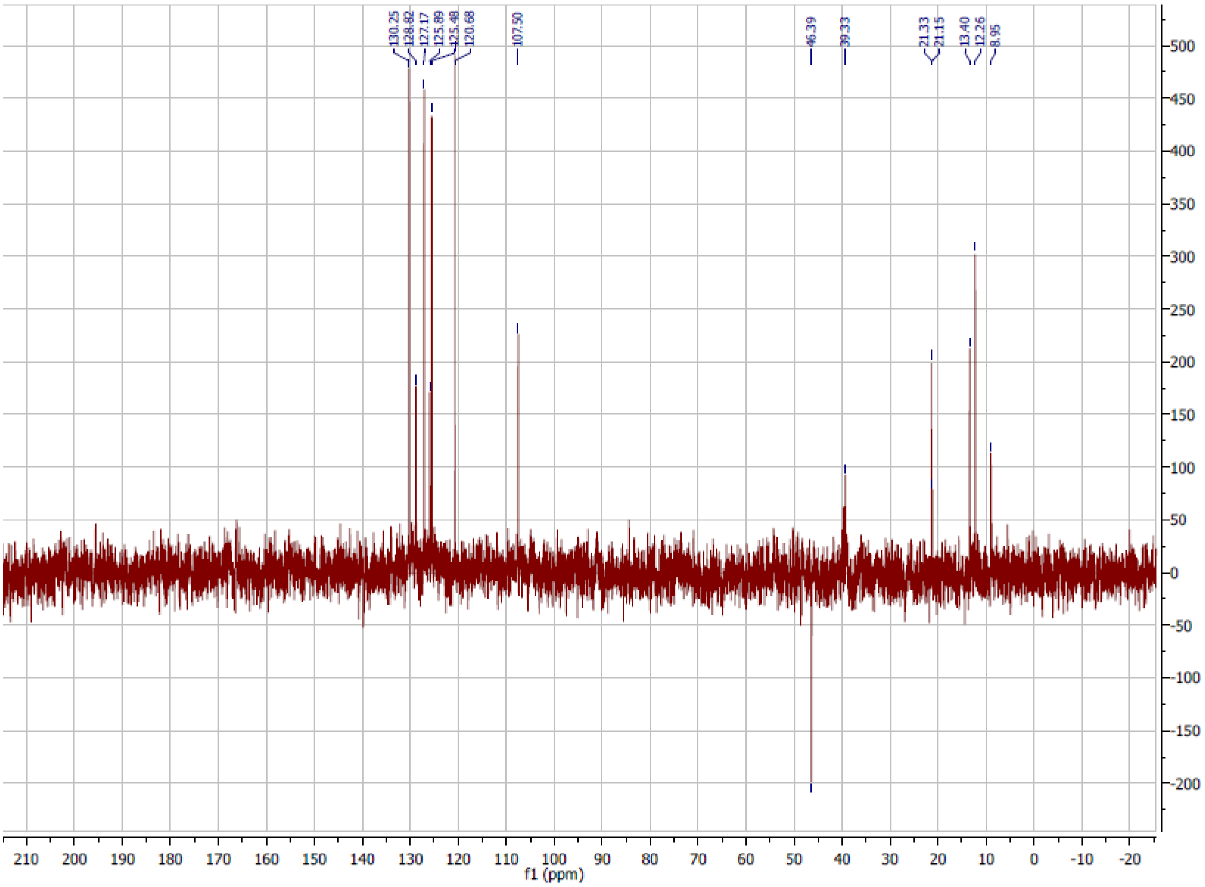

NMR spectra of derivative N-[4-(3,5-dimethylpyrazol-1-yl)phenyl]-4-nitrobenzenesulfonamide (2B)

1H

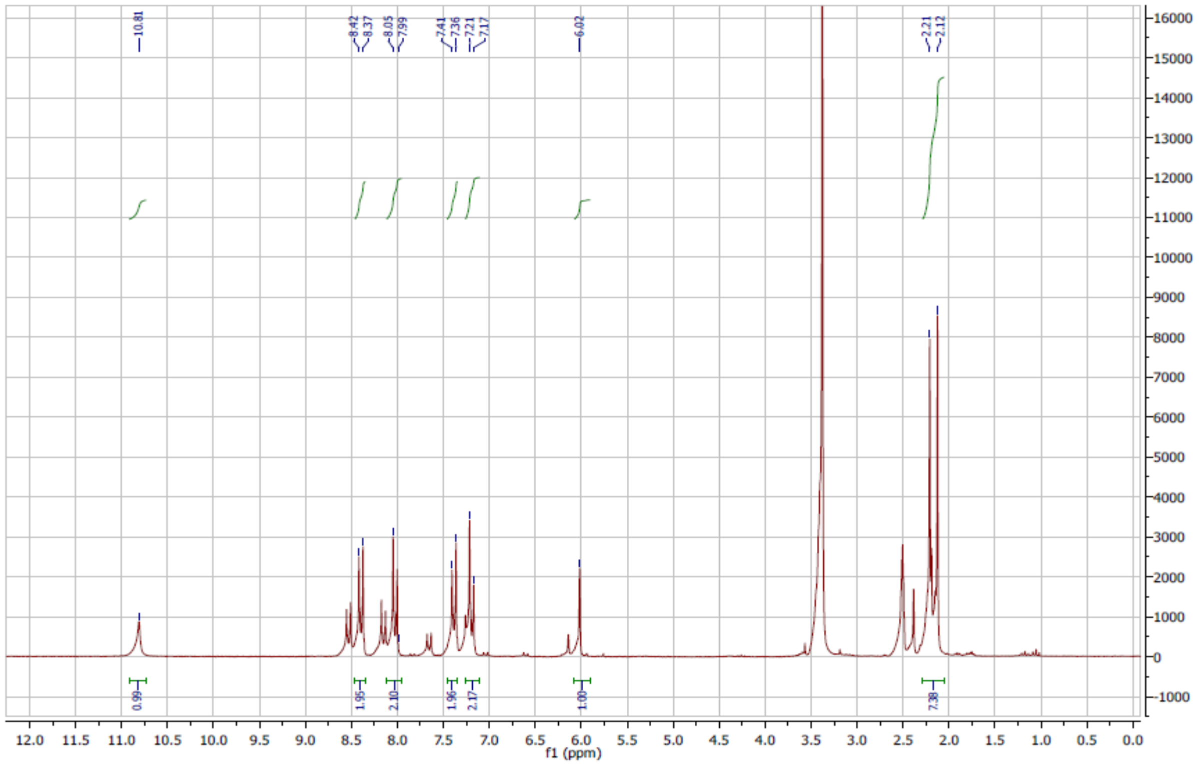

13C

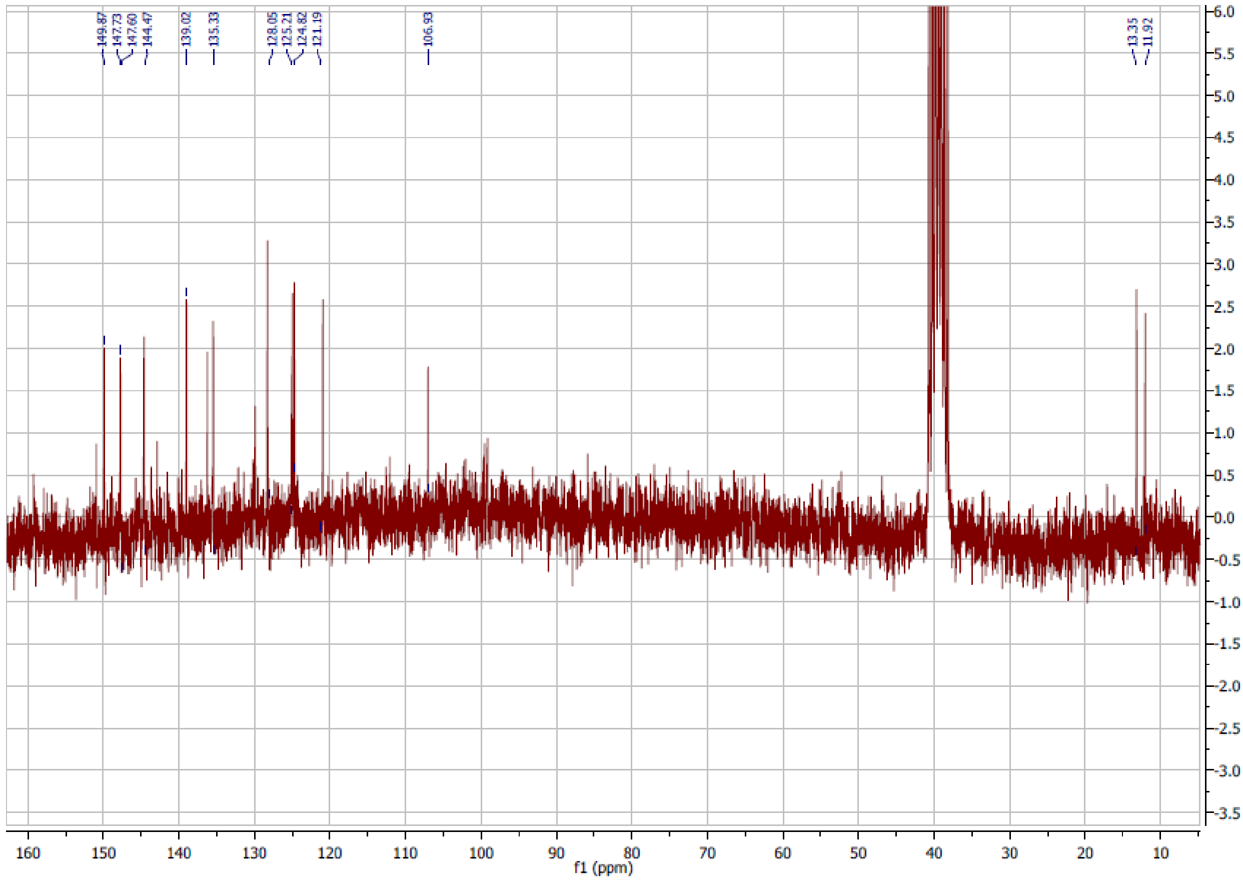

NMR spectra of derivative N-[4-(3,5-dimethylpyrazol-1-yl)phenyl]-4-aminobenzenesulfonamide (2C)

1H

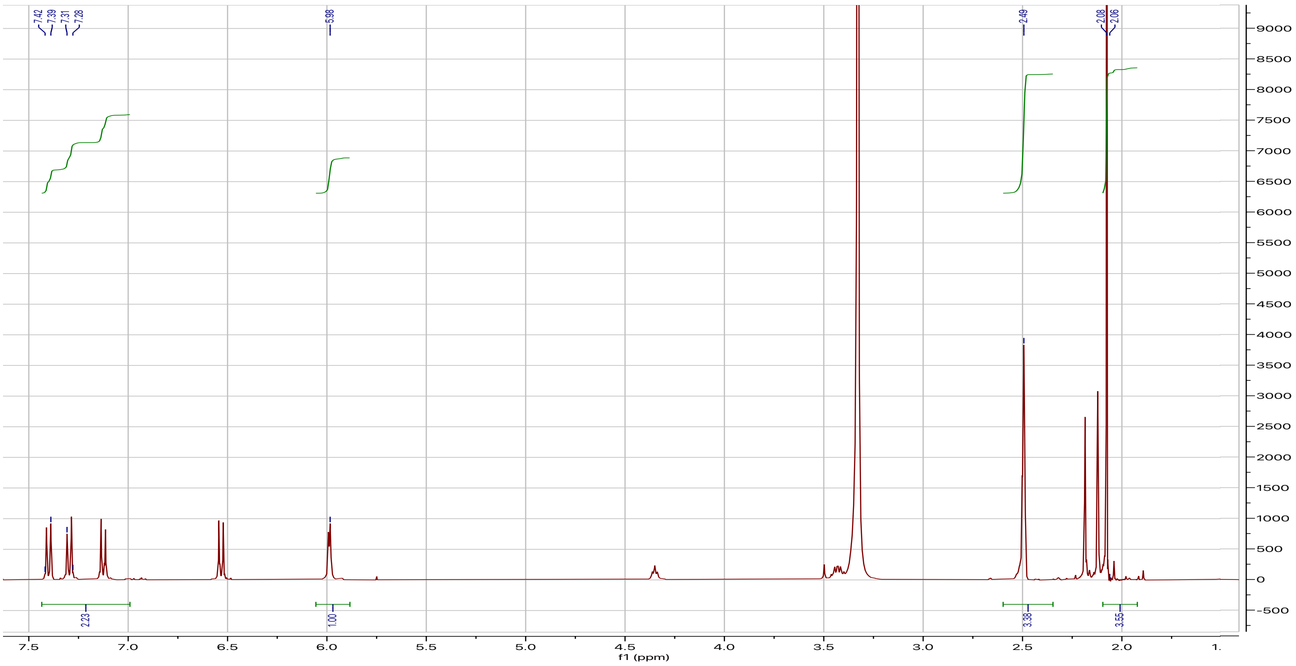

13C

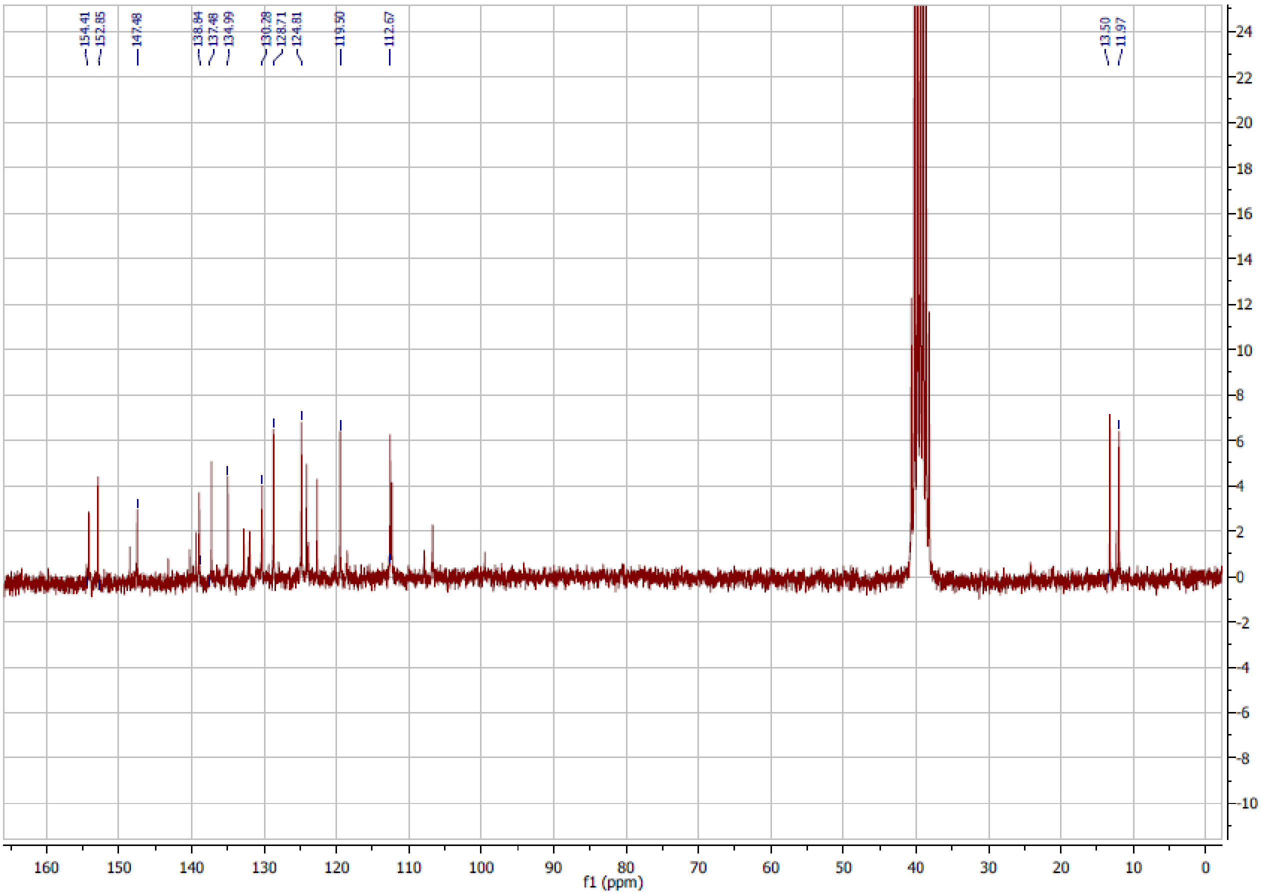

Supplement: Supplementary file 1 [file pharmaceuticals-15-00082-s001.zip › pharmaceuticals-1520201-supplementary.pdf]
